# Effects of tobacco product use on oral health and the role of oral healthcare providers in cessation: A narrative review

**DOI:** 10.18332/tid/157203

**Published:** 2023-01-25

**Authors:** Sangeeta Gajendra, Scott McIntosh, Sucharu Ghosh

**Affiliations:** 1 Eastman Institute for Oral Health, University of Rochester, Rochester, United States; 2 Department of Public Health Sciences, University of Rochester, Rochester, United States; 3 Arthur A. Dugoni School of Dentistry, University of the Pacific, San Francisco, United States

**Keywords:** pharmacotherapy, dentist, tobacco cessation, brief intervention, oral health

## Abstract

Tobacco use has detrimental effects on health, including oral health. The emergence and increasing popularity of newer tobacco and nicotine products make tobacco use one of the major public health problems in the world. Tobacco use increases the risk of oral diseases such as oral cancer, oral mucosal lesions, periodontal disease, and dental caries, among many other oral diseases and conditions. The dental office is an excellent venue for providing cessation intervention. However, there is a lack of knowledge and training in tobacco use prevention among dental professionals. More efforts are needed for smoking cessation interventions in the dental office. Smoking cessation interventions provided by oral healthcare providers include brief educational, behavioral, and pharmacological interventions. This review provides an overview of the ill effects of tobacco use on oral health and the role of oral healthcare providers in managing and preventing tobacco dependence.

## INTRODUCTION

The World Health Organization reported that 23.6% of the global adult population (aged ≥15 years) were current tobacco users in 2018, down from 33.3% in 2000 and projected to decline further to 20.9% by 2025^1^. In the United States in 2018, while an estimated 20% of US adults currently used any tobacco product, 13.7% of US adults (34.2 million people) were current cigarette smokers^2^. From 1965 to 2017, the prevalence of current smoking declined from 52.0% to 15.8% (relative percent change: 69.6%) among men and from 34.1% to 12.2% (relative percent change: 64.2%) among women.

Tobacco and tobacco-related products recently were found to have been used as far back as 12300 years ago^3^. Cigarette smoking is the most popular method of using tobacco. While each cigarette contains 10–14 mg of nicotine, 1–1.5 mg is absorbed into the body when smoked^4^. Tobacco addiction is driven by nicotine, which is the primary reinforcing component of tobacco. Nicotine is generally delivered through the skin, lungs, and mucous membranes.

Smoked tobacco, and in particular, cigarette smoking, is the most popular method of using tobacco. Smoked tobacco is the most common method for nicotine delivery. Smoked tobacco is available in various forms, such as cigarettes, cigars, pipes, bidis, hookah, and others^5^. According to the American Lung Association, a burning cigarette produces more than 7000 chemicals, of which 69 are carcinogens^6^.

Smokeless tobacco is an umbrella term, which includes chewing tobacco, dry snuff, moist snuff, Swedish-style snus, betel quid, gutkha, zarda, toombak, and other products^5^. These products are generally made from a mixture of tobacco, nicotine, sweeteners, abrasives, salts, and chemicals, and contain a mix of 4000 chemicals, more than 30 of which are known carcinogens^7^. Smokeless tobacco delivers 3–4 times more nicotine than smoked tobacco. The amount of nicotine in 8–10 chews/dips per day is equivalent to 30–40 cigarettes per day^8^. Studies from North America, Sweden, and South Asia have linked smokeless tobacco use with pancreatic cancer, oral cancer, cardiovascular, and other diseases^9-12^. Swedish snus, a steam-pasteurized form of tobacco, contains a lower amount of nitrosamine than traditional products and has been promoted as a potential harm-reduction product. Levy et al.^13^ estimated low nitrosamine smokeless tobacco (LNST) to be 90% less hazardous than cigarette smoking and promoting LNST could reduce smoking prevalence by 1–3%. However, simulation models failed to show any significant public health benefits of promoting smokeless tobacco^14^.

Other forms of non-cigarette tobacco include nicotine products like nicotine-containing medications or nicotine replacement therapy (NRT) (examples are transdermal patches, gum, lozenge, sublingual tablet, inhaler, and nasal spray) and electronic nicotine delivery systems (ENDS) or e-cigarettes^5^. Transdermal patch is a slow sustained-release form of nicotine delivery. Other products like gum, nasal spray, oral inhaler, and tablets, are acute dosing forms of nicotine. They provide general craving relief and breakthrough craving relief with the immediate release of nicotine^15^. E-cigarette, a non-combustible tobacco product, comes in several formats resembling for example traditional cigarettes, pens, or USB flash drives using an e-liquid that may contain nicotine and various flavorings, propylene glycol, vegetable glycerin, and other ingredients. The device generates an aerosol that the user inhales. According to the National Youth Tobacco Survey 2021, an estimated 2.06 million youths in the US reported using e-cigarettes within the past 30 days (current use) with 84.7% using flavored e-cigarettes, including 85.8% of high school users and 79.2% of middle school users^16^. Analysis of the National Health and Nutrition Examination Survey (NHANES) for the years 2015–2016 found the weighted prevalence of every use of e-cigarettes among adults (>18 years) was 20%. Compared with adults aged ≥55 years, odds of e-cigarette use were 4.77 times (95% confidence interval, CI: 3.63–6.27) higher among those aged 18–34 years and 2.16 times (95% CI: 1.49–3.14) higher among people aged 35–54 years^17^. The use of e-cigarette use has been associated with respiratory illnesses and other health effects^18^. The evidence base for the harms caused by e-cigarette liquids, their flavors, and their intensity of use has been established and is continuing to grow^19^.

Currently, 16 million Americans are living with a smoking-related disease. In addition to the human costs, smoking places a significant financial burden on US citizens, as smoking-attributable healthcare spending exceeds $170 billion per year^20^. Clearly, there is a strong evidence base supporting smoking as a risk factor for non-communicable diseases such as cardiovascular diseases and cancer^21^. Evidence has linked smoking with lung diseases as well as impacting the immune system and increased susceptibility to infections. Smoking, including e-cigarette use, increases the risk and severity of pulmonary infections because of structural damage to the upper airways and a decrease in pulmonary immune function^22^.

The evidence is also strong and growing regarding the association between smoking and infectious diseases, including increasing the prevalence of HIV, tuberculosis, and COVID-19 due to the alteration of the structural, functional, and immunologic host defenses^23^. For example, the novel coronavirus SARS-CoV-2 that causes COVID-19 affects the respiratory system from mild to severe respiratory symptoms. A recent systematic review and meta-analysis of 40 studies concluded that there is an increased risk of severe COVID-19 disease among current smokers and former smokers compared to non-smokers (OR=1.58; 95% CI: 1.16–2.15, p=0.004; and OR= 2.48; 95% CI: 1.64–3.77, p<0.001). Furthermore, the study found an increased risk of death among COVID-19 patients who are current or former smokers (OR=1.35; 95% CI: 1.12–1.62, p=0.002; and OR=2.58; 95% CI: 2.15–3.09, p<0.001)^24^. Another systematic review of 73 articles and 863331 patients found a significant association between smoking and mortality among COVID-19 patients with a relative risk of 1.19 (95% CI: 1.12–1.27)^25^.

For oral healthcare providers, it is imperative to keep abreast of the latest research on the general and oral health effects of smoked and smokeless tobacco and nicotine products. This can be translated into clinical practice to successfully deliver counseling and tobacco use cessation strategies in the dental office. The purpose of this study is to provide a practical review of the literature and discussion of the effects of tobacco-related products on oral health and the role of oral healthcare providers in preventing tobaccorelated illness.

## DEVELOPMENTS

An electronic search was performed between October 2021 and February 2022. Electronic databases including PubMed, EMBASE, and Google Scholar were searched for systematic reviews, controlled clinical trials, and observational studies using specific keywords. Articles that reported the ill effects of tobacco on oral health (including dental, periodontal, mucosal, salivary glands, implants, and oral cancer) and tobacco cessation interventions used by oral health providers (including educational, behavioral, and pharmacological) were included. Searches were also conducted of websites of leading national organizations such as the American Dental Association, the Centers for Disease Control and Prevention, the US Food and Drug Administration, the National Cancer Institute, the Office of Disease Prevention and Health Promotion, the National Institute on Drug Abuse, the National Cancer Institute, the Agency for Healthcare Research and Quality, the American Lung Association, and the American Psychiatric Association.

### Effects of tobacco-related products on oral health

In addition to associations between tobacco product use and many diseases, cigarettes, smokeless tobacco (e.g. chewing tobacco and snus), and other tobacco uses cause specific oral health issues such as oral cancer, oral mucosal lesions, periodontal disease, implant failure, salivary gland hypofunction, dental caries among many other oral diseases and conditions.

### Oral cancer

Oral cancer is the eighth most common cause of cancer-related mortality in the world. An estimated 54010 new cases were reported in the US in 2021with a 5-year relative survival rate of 66.9% from 2011 to 2017^26^. Oral squamous cell carcinoma (OSCC) accounts for 80–95% of all oral cancers^27,28^. Tobacco, smoked or smokeless, causes oral squamous cell carcinoma (OSCC)^29^. Cigarettes and other combusted tobacco products are dangerous nicotine delivery devices that contain a complex mixture of tumor promoters, co-carcinogens, and various toxicants that exacerbate the effects of the carcinogens^30^. In a narrative review of 32 selected articles, Jiang et al.^29^ proposed a plausible carcinogenic pathway attributing tobacco as the major risk factor for OSCC. Tobacco may cause epigenetic alteration of oral epithelial cells and inhibit multiple systemic immune functions of the host. Its toxic metabolites may also cause oxidative stress on tissues releasing reactive oxygen species that can damage, cause mutations and induce OSCC. Studies of e-cigarette chemicals in vaping liquid have also been shown to cause oxidative stress on tissues^31^, including oral tissues^32^. Oral cancer is the eighth most common type of cancer and one-third of oral cancer-related mortality in the world is attributable to tobacco smoking^33^. In a meta-analysis of 15 case-control studies, Sadri et al.^34^ found that smokers are 4.65 (95% CI: 3.19–6.77) times more likely to have oral cancer. Oral cancer related to smokeless tobacco is most prevalent in Asia and Africa. A meta-analysis of 12 systematic reviews found that the estimated risk for oral cancer ranged from 1.36 to 7.90 with a higher risk for the South-East Asia Region (4.44–7.90)^35^. Another systematic review found tobacco chewing increased the risk of oral cancer by 4.7 (95% CI: 3.1– 7.1) times and paan (betel leaf and areca nut) with tobacco increases the risk by 7.1 (95% CI: 4.5–11.1) times^36^.

Exposure to secondhand smoke or passive smoking is a risk factor for several adverse health effects. A systemic review of 1179 cases and 5798 controls found that people exposed to secondhand smoke (SHS) are 1.51 (95% CI: 1.20–1.91) times more likely to have oral cancer. When the duration of exposure was more than 10–15 years, the odds ratio increased to 2.07 (95% CI: 1.54–2.79). This systematic review and meta-analysis supports a causal relationship between SHS and oral cancer and provides guidance to develop policy and appropriate prevention programs^37^.

Smoking and alcohol have a synergistic effect on oral cancer development. A systematic review of 33 articles by Mello et al.^38^ concluded the following synergistic effects: alcohol and tobacco smoking (OR=4.74; 95% CI: 3.51–6.40), alcohol and smokeless tobacco (OR=7.78; 95% CI: 2.86–21.14), and alcohol, tobacco smoking, and smokeless tobacco (OR=16.17; 95% CI: 7.97–32.79) increased the risk for oral cancer. Smokers who are at high risk for cancer need to be identified early to prevent the onset of this disease.

### Oral mucosal lesions

A strong association has been found between tobacco use and mucosal lesions such as leukoplakia, smokeless tobacco keratosis at the site of tobacco placement, nicotinic stomatitis, smoker’s melanosis^39^ and erythroplakia^40^. While some of the oral mucosal lesions are non-malignant, it is necessary to further investigate leukoplakia associated with tobacco with a biopsy for the presence of epithelial dysplasia or carcinoma. About 3 to 6% of leukoplakias undergo malignant transformation, with this frequency increasing with longer follow-up periods^41^. Epithelial dysplasia may appear clinically white or red due to hyperkeratosis or epithelial atrophy, respectively. Epithelial dysplasia that involves the full thickness of epithelium but does not invade the connective tissue is termed carcinoma in situ^39^. Heavy smokers may also have a condition called the black hairy tongue. The dorsal surface of the tongue has a hair-like appearance due to hypertrophy of filiform papillae and retardation of the normal rate of desquamation^39^. Assessment of oral mucosal lesions along with the risk factors is important for their appropriate management.

### Periodontal disease and dental implants

Periodontal disease is a preventable disease in which tobacco use is considered the strongest modifiable risk factor. As early as the 1940s, studies have found a relationship between tobacco use and periodontium. Smokers have higher gingival recession, tooth loss, and pocket depths. compared to non-smokers^42^. A recent systematic review found that tobacco smoking increases periodontitis by 85% (RR=1.85; 95% CI: 1.5–2.2)^43^. Tobacco smokers display an increased gingival microvascular density with considerable gingival inflammation, suppressed angiogenesis due to local immune suppression, and oxidative stress leading to periodontal disease and increased risk of complications^44^. In a cross-sectional pilot study^32^, saliva and gingival crevicular fluid samples were collected from cigarette smokers (CS), e-cigarette smokers (EC), dual smokers (DS), and non-smokers (NS). The samples were analyzed to compare biomarkers of inflammation, oxidative stress, anti-inflammatory lipid mediators, tissue injury and repair, and growth factors with immunoassay (enzyme-linked immunosorbent assay and Luminex) in the four groups. Levels of inflammatory mediators and oxidative stress were statistically significantly higher in EC versus NS as well as in DS versus EC^32^. Additionally, smokers had approximately 80% higher risk of periodontitis than quitters (RR=1.79; 95% CI: 1.36–2.35) and never smokers (RR=1.82; 95% CI: 1.43–2.31)^45^. On a positive note, a systematic review found that those who quit after a smoking cessation program have a similar risk of periodontal disease as those who never smoked (RR=0.97; 95% CI: 0.87–1.08). This finding was supported by a meta-analysis of longitudinal studies that found former smokers and never smokers have a similar risk of tooth loss. However, smokers have 2.6 (95% CI: 2.29–2.96) times more risk of tooth loss^46^.

The deleterious effects of tobacco smoking on dental implants have been extensively studied and multiple systematic reviews were published in the last few years. Smoking has been attributed as the primary patient-centered risk factor for endosseous implant loss^47^. The implant failure rate was found to be higher among smokers^48^. A systematic review found a dose-response effect between cigarette smoking and implant failure. The patients who smoked more than 20 cigarettes per day had a significantly higher risk of implant failure than non-smokers^49^. Negative effects of smoking might be through both systemic and local routes. The heat from smoking and its toxic by-products such as nicotine, carbon monoxide, and hydrogen cyanide may impair healing. In addition, arteriolar vasoconstriction and decreased blood flow may affect the success of surgical procedures involving implants^50^. Smoking cessation may be an effective strategy to improve the success rate of implants.

### Effects on salivary glands

Even though the available literature is sparse, tobacco smoking has been associated with increased sialolithiasis (stones within salivary ducts) formation and decreased salivary flow rate. A cross-sectional study of 947 cases and 3788 controls, found a statistically significant association between smoking and sialolithiasis with an odds ratio of 1.31 (95% CI: 1.08–1.59). Multiple studies have concluded that smoking is associated with thick saliva with reduced salivary flow compared to non-smokers^51^. Smoking has been found to decrease saliva pH^52^ and alter secretory immunoglobulin A (sIgA) levels^53^. All these factors could lead to an increase in periodontal disease and dental caries.

### Dental caries

Dental caries is a multi-factorial disease. Its etiology is mostly related to poor eating habits, oral hygiene, and compliance with treatment. Studies have shown a relationship between smoking and cariescausing bacteria^54^. Nicotine may cause an ecological imbalance and promote colonization and metabolism of *Streptococcus mutans*, a significant bacterium contributing to dental caries. Smoking influences saliva by lowering the buffer capability, altering its chemical agent and bacterial components^54^, and reducing salivary flow rate^55^ thereby promoting the formation of a caries-susceptible environment^54^.

Several epidemiological studies have linked tobacco use and increased dental caries prevalence^54,56,57^. Findings from the National Health and Nutrition Examination Survey (NHANES) from 2011 to 2016 found that 40–50% of adult smokers aged 20–64 years have untreated dental decay, specifically among non-Hispanic Black, Mexican American, or poor and near-poor, combined as well as those who had a high school education or lower. The prevalence was twice that of adults who were non-Hispanic White or notpoor, who had more than high school education, and who had never smoked. Smokers aged ≥65 years are twice as likely to have untreated caries than those who never smoked^56^. Studies from Italy and Finland have shown smokers have higher decayed, missing, filled teeth (DMFT) scores than non-smokers^58,59^. Two systematic reviews, published in 2013 and 2019, have found a positive correlation between tobacco smoking and increased dental caries. However, both studies concluded that the present evidence is poor and there is a need for more prospective studies^60,61^. A 3-year epidemiological surveillance open cohort study of 22009 patients found that 36.6% had dental caries with smoking as a risk indicator for dental caries with an odds ratio of 1.84 (95% CI:1.64–2.07). The study also found that smoking prevention can lead to a 7% potential reduction in dental caries^57^. Thus, dental caries and other oral health findings attributed to tobacco use should be part of the discussion for quitting.

### Impact of socioeconomic factors on the use of tobacco-related products and oral health

Smokers are at increased risk for oral diseases. With the advent of new combustible, noncombustible, and electronic tobacco products being available in the US, it is imperative to determine the impact of socioeconomic factors on tobacco product use and oral health. This will help in designing targeted interventions in concert with the regulation of tobacco products to reduce tobacco-related diseases^62^. Profound oral health disparities are seen in specific subpopulations in the US. Untreated decay, tooth loss, and periodontal disease are disproportionately higher among racial and ethnic minorities, low-income individuals, those with limited education, with public dental insurance or without any dental insurance, and smokers^63^. Smokers have lower rates of dental care utilization compared to non-smokers^64,65^. Participants of the OralHealth4Life trial, eligible callers to the Louisiana, Nebraska, and Oregon state tobacco quitlines, mostly low-income individuals with high school or lower education, cited cost and no dental insurance as barriers to receiving dental care. After controlling for these financial factors, the following baseline characteristics were significantly associated with a higher likelihood of dental care utilization at 6 months: higher motivation (relative risk, RR=2.16) and self-efficacy (RR=1.80) to visit the dentist, having a disability (RR=1.63), having a higher education level (RR=1.52), and having perceived gum disease (RR=1.49)^66^. Data from a large, populationbased case-control study of oral cancer risk factors conducted in four areas of the US found that various environmental or lifestyle determinants of oral cancer may contribute to the higher oral cancer rates in Blacks than in Whites in the US, but that patterns and risks associated with alcohol consumption, particularly among current smokers, are the most important contributors to the excess risk in Blacks^67^. Indeed, the use of tobacco-related products contributes to health disparities and inequalities and needs to be addressed in cessation programs.

### Role of oral healthcare providers in cessation

Quitting tobacco use greatly reduces the risk of developing many diseases^20^. Dependence on tobacco or nicotine is a chronic condition that warrants interventions by all healthcare providers, including oral healthcare providers. Patients need multiple attempts to successfully achieve abstinence. A study by Babb et al.^68^ found that 68% of adult smokers wanted to stop smoking, 55% made a past-year quit attempt, and about 7% quit smoking. The study also found that 57.2% had been advised by a health professional to quit, and 31.2% used cessation counseling and/or medication when trying to quit. The goal for Healthy People 2030 is to increase past-year attempts to quit smoking in adults from 56% in 2018 to 65.7% and increase successful quit attempts from 8.3% in 2018 to 10.2%^69^. Understanding the role that barriers play in quitting tobacco use is helpful. Cessation programs need to address barriers to improve the success rates for quitting. A two-wave survey to explore self-reported barriers to quitting among young adult smokers found that low SES smokers reported several barriers. The risk of gaining weight was statistically significant between low SES and high SES. Other barriers like the cost of classes or programs, craving or withdrawal from nicotine, loss of a way to handle stress, and friends’ smoking were prevalent but not significant^70^.

The dental office is an excellent venue for providing cessation intervention as 46.6% of patients who smoke make an annual visit to the dentist^71^. There are more than 200000 professionally active dentists in the US^72^. On average, a dentist sees more than 68 patients per week (including hygiene appointments)^73^ and the hygienist sees approximately 45–50 patients per week^74^. The entire dental team needs to be involved in tobacco cessation, including dental hygienists and assistants. Patients tend to have a better rapport with dental hygienists and pay more attention to their oral health educational messages. Integration of tobacco cessation by the electronic health record system with automated clinical reminders is a useful tool. A recent study reported that although dental professionals ask for and document patient tobacco use (hygienists: 80%; dentists: 73%), they did not frequently assist in tobacco cessation (hygienists: 27–49%; dentists: 10%–31%). The findings from this study suggest that efforts to engage dental professionals in tobacco cessation should prioritize increasing dental providers’ relevant knowledge, skills, and sense of professional responsibility^75^. A randomized controlled trial of a smoking cessation intervention (combination of brief counseling using the 5As model and NRT) delivered by dental hygienists found that a statistically higher percentage of intervention participants had a quit attempt of at least 1 week at 3 months (15% intervention group vs 9% controls) and 6 months (10% intervention group vs 5% controls). This feasibility study has shown the potential that trained dental hygienists could have in delivering smoking cessation advice^76^.

A study involving analysis of national data NHANES (2015–2018) in which 1024 adult respondents who were current or former smokers who quit smoking within the past 12 months, and reported a dental visit within the past 12 months, were included in the study. Among the study subjects, only 44.6% received smoking-cessation advice from a dental care professional. The authors found no significant association between smoking-cessation advice and any attempt to quit smoking. Although the respondents who received smoking-cessation advice reported 18% more quit attempts, the advice was not associated with abstinence of 6 months or longer. Thus, receiving smoking-cessation advice from a dental care professional was associated with more attempts to quit smoking^77^.

Based on an extensive review of the existing scientific literature, the most recent Surgeon General’s Report of 2020 on smoking cessation^20^, concluded that proven smoking cessation treatments are widely available today. However, the reach and use of existing smoking cessation interventions remain low. There are gaps in the utilization of programs, policies, and resources that can improve cessation rates and help smokers quit. The report also stated that the evidence is sufficient to infer that the development and dissemination of evidence-based clinical practice guidelines increase the delivery of clinical interventions for smoking cessation. Thus, more efforts are needed for smoking cessation interventions in the dental office.

Tobacco cessation in oral health settings is both feasible and effective. Incorporating behavioral interventions for tobacco cessation within routine oral examinations help tobacco users quit^78^. Tobacco cessation interventions provided by oral healthcare providers can be classified into three categories, which include brief educational, behavioral, and pharmacological interventions. The interventions can be used alone or in combination^78^.

### Brief educational interventions

Brief interventions involve raising awareness about the harmful effects of tobacco products on general health. The 5As model is the most recognized and widely accepted framework for brief smoking cessation intervention. This model was developed by the United States Department of Health’s Clinical Practice Guideline in 2000 and is based on the transtheoretical model of behavior change, or the stages of change model^79^. It proposes that smokers should be given a brief intervention to address smoking at every health consultation. This model is based on five strategies:

1. Ask about the duration of tobacco use, amount, and type of tobacco use (1 min).
2. Advise all smokers to quit in a clear, strong, and personalized manner (30 sec).
3. Assess the subject’s willingness to quit smoking using the Prochaska Stages of Change model^80^. Assess the patient’s willingness to quit within the next 30 days. If a patient is willing to try to quit within the next 30 days, move to the Assist step. If not, use the 5Rs to try to increase their motivation (30 sec).
4. Assist the subject with a plan for quitting and set a quit date (3–5 min).
5. Arrange a follow-up one month after the quit date either in person or via telephone (5 min).

For patients who are not ready to make a quit attempt in the next 30 days, the oral health providers may use the 5Rs strategy^79^:

1. Relevance – encourage the patient to indicate why quitting is personally relevant.
2. Risks – ask the patient to identify potential negative consequences of tobacco use.
3. Rewards – ask the patient to identify the potential benefits of stopping tobacco use.
4. Roadblocks – ask the patient to identify barriers or impediments to quitting.
5. Repetition – the motivational intervention should be repeated every time an unmotivated patient has an interaction with a clinician.

Even though the 5As model remains the most accepted model for brief intervention, there are limitations such as lack of time and expertise^81^. A recent systematic review^82^ was conducted to determine if dental professionals could help people to stop using tobacco by offering them advice and support. All studies used behavioral programs aimed to boost motivation and offer advice to help people stop using tobacco. The study found that behavioral programs involving dental professionals and NRT or e-cigarettes probably help more people to stop smoking. On average, 74 out of 1000 people stopped compared with 27 out of 1000 people who did not receive behavioral support. However, the authors were moderately confident about the benefit of support from dental professionals plus NRT or e-cigarettes.

To overcome the common barriers of the 5As model, a three-step model was proposed, Ask, Advise, and Refer: Ask every patient about tobacco use; Advise all tobacco users to quit; and Refer tobacco users to nationally available tobacco cessation quitlines^79^. The New South Wales (NSW) Oral Health Promotion Network developed an abbreviated 3As model: Ask about and record smoking status; Approach smokers about their interest in quitting (using the stages of change model); and advise of NSW Quitline and refer to appropriate services^81^.

### Behavioral interventions

Behavioral interventions aim to motivate, guide, and psychologically assist smokers to quit^83^. A specific taxonomy for classifying Behavior Change Techniques (BCT) targeted to smoking cessation is called Behavior Change Techniques Taxonomy for Smoking (BCTTsm)^83^. This taxonomy includes 44 BCTs and classifies them into four groups^84^:

1. Directly addressing motivation (e.g. boosting motivation and self-efficacy);
2. Maximizing self-regulatory capacity and skills (e.g. facilitating relapse prevention and coping);
3. Promoting adjuvant activities (e.g. advice on stopsmoking medication); and
4. Supporting other BCTs (e.g. focus on the delivery of the intervention).

Hartmann-Boyce et al.^85^ conducted a meta-analysis of 33 Cochrane Reviews and found that behavioral interventions for smoking cessation such as any form of counseling along with guaranteed financial incentives provided the most motivation to quit for six months or longer^85^. Another systematic review concluded that goal setting has a unique effect across a range of behaviors and it is particularly effective when the goal is in a group, difficult, and set publicly^86^. A recent Cochrane Review found that behavioral support provided by dental professionals is beneficial for smoking cessation at six months compared to brief or no intervention. Multiple-session programs have a higher quit rate than single-session programs^82^. Systematic reviews did not find any harmful effects as a result of behavioral intervention by the dentist^82,85^.

A systematic review^84^ published in 2021 evaluated BCTs used for tobacco cessation in dental practices and their effects on intervention. They found that 16 out of 44 BCTTsm were used in general and 2–11 BCTs were included in the interventions. The authors did not find any association between the number of BCTs and intervention effectiveness. The most commonly used BCTs are: facilitating goal setting, offering/directing towards appropriate written materials, assessing current readiness and ability to quit, assessing current and past tobacco-use behavior, advising on/facilitating the use of social support, providing feedback on current behavior and advice on stop-tobacco medication.

Telephone quitlines are a cost-effective^87^, evidenced-based approach for providing behavioral counseling across large geographical areas and populations. The first quitline was established in California in 1992 and is now available in all 50 US states, the District of Columbia, Guam, and Puerto Rico. Quitline services are also available through the National Asian Smokers’ Quitline. Since its launch in 2004, 1-800-QUIT-NOW has received more than 10 million calls. Quitlines not only offer counseling but also offer free nicotine replacement therapy^88^. A United States Public Health Service-sponsored Clinical Practice Guideline meta-analysis in 2008^79^ found that quitlines increased overall quit rates by about 60% when compared to minimal counseling, no counseling, or self-help. The guidelines also reported that healthcare providers are more likely to provide smoking cessation interventions if state quitlines are conveniently available as a referral source. A systematic review of smokeless tobacco cessation intervention studies conducted globally found regular telephone support/quitlines also proved to be beneficial^89^.

### Pharmacotherapy

Smoking cessation advice for even a few minutes increases long-term smoking abstinence rates by 5%, which can be increased by 50–70% with the use of adjunctive pharmacotherapy, e.g. nicotine replacement therapy for withdrawal symptoms^90^. The US Preventive Services Task Force found a substantial benefit of FDA-approved pharmacological and behavioral interventions, both individually and in combination to increase smoking cessation among non-pregnant adults^91^. Pharmacological interventions help to reduce withdrawal symptoms associated with cessation attempts by curbing nicotine cravings^92^. They include nicotine replacement therapies (NRTs), varenicline, cytisine, and bupropion SR.

*Nicotine replacement therapy*

Among the various pharmacotherapies, nicotine replacement therapy (NRT) is the most commonly used. Nicotine is a chemical that acts as an agonist of nicotinic acetylcholine receptors in the ventral tegmental area of the brain. When stimulated, the nicotinic receptors release dopamine in the nucleus accumbens leading to a sense of reward. NRT products contain pure nicotine and aim to reduce the desire for smoking by increasing nicotine levels in the bloodstream through sources other than cigarette smoke^92^. Several types of NRT products are available in the market.

Nicotine gum, an easily accessible NRT product, is prescribed for 6–12 weeks for a maximum of 6 months. Patients are advised to chew intermittently for 30 minutes and then place it in the oral vestibule for transmucosal absorption. After 2–3 months the dose is tapered or the chewing time is gradually decreased^93^. Generally, gums with 4 mg of nicotine have a higher success rate than gums with 2 mg of nicotine. In a recent randomized clinical trial, Hansson et al.^94^ found that 6 mg nicotine gums provide a faster and greater reduction of urges than 4 mg gums. Some side effects of nicotine gum include soreness, hiccups, dyspepsia, and jaw pain^95^.

Nicotine lozenges come in two sizes (regular and mini) and two strengths (2 mg and 4 mg). It is recommended to use 1 lozenge every 1–2 hours for the first six weeks of the quit attempt^96^. The dose is then tapered and then stopped. Chronic overuse of NRT, specifically nicotine lozenges are associated with hyperkeratotic lesions^97^. Oral healthcare providers need to be aware of the oral effects associated with NRT products.

Nicotine patches are transdermal patches that release nicotine slowly over time. Two forms of patches are available which can be worn either for 16 hours or 24 hours. The 16-hour patches are available in the form of 5, 10, and 15 mg doses, and the 24-hour patches are available in 7, 14, and 21 mg doses^98^. In a randomized placebo-controlled trial, Schnoll et al.^99^ found that the 24-week patch treatment may be more effective in reducing the chances of relapse and the weight gain than the 8-week patch treatment. Insomnia and local skin irritations have been reported as side effects of nicotine patches^98^.

Nicotine inhalers mimic cigarettes and consist of a mouthpiece and a plastic cartridge^98^. Each inhaler contains 10 mg of nicotine which can be sprayed in the mouth without touching the lips^93^. Nicotine nasal sprays were designed for the rapid delivery of nicotine. Multiple studies have shown that nasal sprays can deliver nicotine more rapidly than other NRT products^98^. CDC recommends patients take 1–2 doses per hour with a maximum of 40 doses per day. However, in a randomized trial, Rubinstein et al.^100^ did not support the use of nasal sprays as an adjunct to counseling for adolescent smokers due to unpleasant adverse effects, poor adherence, and consequent lack of efficacy.

Oral healthcare providers can use the Fagerström scale to assess the severity of tobacco addiction. On this scale, patients are asked 6 scored questions and based on the total score, NRT can be prescribed^101^. Oral health providers can use various methods to help their patients with NRT. The NRT sampling (NRTS) method is a short starter course of NRT prescribed to all eligible smokers regardless of their motivation to quit^102^. Carpenter et al.^103^ found that a free 2-week starter kit of NRT (both patch and lozenge) increased quit attempts, use of smoking cessation medications and abstinence, compared to standard care and the effects were consistent despite the smoker’s motivation to quit^103^. NRT products with stain removal or tooth-whitening activity can be useful for oral healthcare providers to show early measurable benefits of smoking cessation^102^. Whelton et al.^104^ found that the tested nicotine replacement gum can help in stain reduction and can help oral healthcare providers motivate those wishing to quit smoking. Oral healthcare providers have a unique opportunity to educate the public about the safety of NRT products by debunking the misconception of the carcinogenic effects of nicotine^102^.

*Varenicline (CHANTIX/CHAMPIX)*

Varenicline is a smoking cessation aid, which is used in combination with education and counseling. Studies have shown varenicline to have more efficacy than bupropion SR and nicotine patches. It acts as a partial agonist of the alpha-4-beta-2 nicotinic acetylcholine receptors and inhibits the activation of the dopaminergic pathway which is linked to the withdrawal syndrome during cessation attempts^105^.

Varenicline comes as a tablet and is only given to adult patients. The therapy starts 1 week before the target quit date with a tapered increase dose (Days 1–3: 0.5 mg once daily; days 4–7: 0.5 mg twice daily; days 8–11: 1 mg twice daily). Patients are advised to take the tablet with a full glass of water after a meal to avoid stomach upset. In patients with renal impairment, a maximum dose of 0.5 twice daily and with end-stage renal disease maximum dose of 0.5 mg once daily is recommended^106^.

Common side effects of varenicline include nausea, insomnia, abnormal vivid dreams, and headaches^105^. It also increases the risk of pancreatitis, and kidney stones and failure. Patients using varenicline should be under close supervision for behavioral changes as there is an FDA-mandated warning for severe psychiatric symptoms including suicidal symptoms^106^. Furthermore, it contains N-nitroso-varenicline impurity which is carcinogenic. However, the health benefits of stopping smoking outweigh the cancer risk from the nitrosamine impurity in varenicline^107^.

*Bupropion SR*

Bupropion has been widely used as an antidepressant. It is also used as a smoking cessation aid. The mechanism of action is not fully understood but it seems that bupropion weakly inhibits norepinephrine and dopamine. Also, it has some action on nicotinic and serotonin receptors^108^.

Bupropion SR tablets can be regular or extendedrelease (12- or 24-hour) and are available from 75 to 522 mg forms. Patients are advised to take the whole tablet once daily with or without a meal. Newly prescribed patients should be closely monitored for behavioral changes as bupropion SR is known to cause suicidal tendencies^108^.

Common side effects of bupropion SR are tachycardia, rhinitis, pharyngitis, insomnia, headache, agitation, dizziness, diaphoresis, weight loss, constipation, dry mouth, nausea, tremor, and blurred vision. More than 10% of the patients suffer one or more side effects^108^.

*Cytisine*

Cytisine is a plant-based alkaloid and has been used in eastern Europe for smoking cessation since 1964. It acts as a partial agonist of alpha-4-beta-2 nicotinic acetylcholine receptors and inspired the development of varenicline^109^. Oral cytisine has a shorter half-life (4.8 vs 17 hours) and treatment course (3.5 vs 12 weeks) than varenicline^110^.

A recent systematic review and meta-analysis found that patients on cytisine had 1.74 (95% CI: 1.38–2.19) times higher successful continuous abstinence at the longest follow-up than those using a placebo^111^. Nausea, vomiting, dyspepsia, upper abdominal pain, and dry mouth were reported as the side effects of cytisine^111^.

*Combination therapy*

Combination therapy of drugs with a distinct mechanism of action or therapeutic properties helps to achieve therapeutic synergism^112^. A meta-analysis of five clinical trials of pooled 2204 patients found that combination therapy was significantly better than monotherapy (p<0.05). The relative risk of abstinence comparing combination with single treatment groups was 1.42 (95% CI: 1.21–1.67), 1.54 (95% CI: 1.19–2.00), and 1.58 (95% CI: 1.25–1.99) at 3, 6, and 12 months, respectively. Primarily, two following types of combination therapy include a combination of different NRTs or NRTs with non-NRT drugs^113^.

First, a combination of NRTs with different pharmacokinetic profiles such as nicotine patch + nicotine gum, patch and inhaler, patch and nasal spray, etc. Withdrawal symptoms can be better managed with combination NRTs as sustained-release NRTs (e.g. nicotine patch) to maintain a stable baseline nicotine level in addition to immediate release NRTs (e.g. gum, spray, inhaler, etc.) that can intermittently increase blood nicotine level^113^. A systematic review including a total of 3200 participants found that combination NRT has a significantly higher quit rate at 6 months or longer than single or no NRT^114^.

Second, a combination of NRT and non-NRT drugs such as bupropion SR and NRT, nortriptyline and NRT, and varenicline and NRT, has proven to be effective. The only FDA-approved combination therapy for smoking cessation is bupropion SR and nicotine patches^113^. In general, studies suggest that bupropion SR in combination with NRT increases the quit rates in the short-term but long-term benefits are insignificant according to the United States Public Health Service Guideline meta-analysis^79^.

### Barriers to smoking cessation interventions

There is a lack of studies focusing on the perceived barriers to oral health providers while providing tobacco cessation interventions. In their systematic review, Carr and Ebbert^78^ cite several barriers across studies they reviewed, including: 61.5% of dentists believe patients do not expect tobacco cessation resources, in spite of the fact that 58.5% of patients felt these resources should be provided; concern for patient resistance; lack of knowledge; lack of time; lack of financial reimbursement; and concern for unsuccessful patient follow-up to tobacco cessation resources. A more recent systematic review^115^, by Goel et al.^116^ in 2020, found that dental practitioners lacked satisfactory knowledge, confidence and training, and were unaware of existing referral pathways to specialist smoking cessation services.

In a study on general dentists in California, Pennsylvania, and West Virginia, the barriers to cessation counseling included: patient resistance (66%); lack of insurance reimbursement (56%); not knowing where to refer (49%); and lack of time (32%). Similar barriers were identified among dental hygienists^75^. The authors found that perceived patient resistance (per the hygienists) and lack of training (per the dentists) were the most cited barriers to providing tobacco cessation. The authors also found that greater confidence and willingness to assist were positively associated with providing assistance in multivariable models, but perceived barriers (e.g. lack of time and remuneration) were not. The authors concluded that greater dental professional engagement in tobacco cessation will require expanding providers’ self-efficacy, perceived professional scope, and motivation and likely will require system and organizational change^75^. A similar study found that dental hygienists reported greater levels of activity and confidence, fewer barriers, and longer consultation times compared to dentists. All participants indicated high rates of advising patients to quit smoking, but low rates of assisting and referring patients^117^. Identifying barriers to tobacco cessation counseling may enhance effectiveness, and should be addressed in the American Dental Hygienists’ Association’s ‘Ask, Advise, Refer’ initiative^79^.

Adequate reimbursement for providing tobacco cessation is essential to incentivize health professionals, including dental professionals to promote tobacco cessation among smokers. Considering the prohibitive costs associated with tobacco-related illnesses, both public (Medicaid and Medicare) and private insurance (individual purchased and employersponsored) should cover tobacco cessation programs. The prevalence of current cigarette smoking is approximately twice as high among adults enrolled in Medicaid (23.9%) as among privately insured adults (10.5%), placing Medicaid enrollees at increased risk for smoking-related disease and death^2^ . There is strong evidence that comprehensive, barrier-free state Medicaid cessation coverage could reduce smoking, smoking-related disease, and healthcare expense among Medicaid enrollees. While all 50 states and the District of Columbia covered some cessation treatments, only 15 states, as of 31 December 2018, covered all nine cessation treatments with some barriers (copayment, prior authorization, counseling required for medications, limits on duration, etc.) in place for some treatments^118^. Both Medicaid and Medicare have started reimbursing dental practitioners for tobacco cessation. New York State in particular has been the front-runner for this^119^. However, a survey of New York State dentists and dental hygienists found that only 26.7% of dentists were aware that dentist smoking cessation counseling is covered and 15.5% of hygienists were aware that hygienist smoking cessation counseling is covered by Medicaid^120^. Among private insurers, adults are more likely to visit a dentist annually for routine preventive care. Thus, coverage for tobacco cessation by private insurance would be beneficial. More studies are needed to assess barriers and facilitators to deliver tobacco cessation interventions successfully in the dental office.

### Implications

Dental treatment involves several visits to the dental office providing multiple opportunities for the dental team to manage and prevent tobacco product use and nicotine dependence. Tobacco product use should be addressed with every patient at every dental visit. Integration of tobacco cessation by the electronic health record system with automated clinical reminders is a useful tool. The evidence is strong for the effectiveness of tobacco cessation involving brief behavioral interventions complemented by pharmacological treatment and referral to state quitlines and quitsites. The entire dental team needs to promote tobacco product cessation to their patients to ensure successful quit attempts.

## CONCLUSION

Tobacco has detrimental effects on oral health. Tobacco users have significantly higher rates of oral cancer, oral mucosal lesions, periodontal disease, dental caries, and implant failure. Dental practice settings provide a unique opportunity in providing tobacco cessation assistance. Oral health providers can refer patients to free evidence-based treatment options such as telephone quitlines, quitsites, and telehealth, and they can use brief educational interventions, behavior counseling, and pharmacotherapy including nicotine replacement therapy. Lack of time and training are the most common barriers faced by dentists while providing tobacco counseling services. Considering the myriad of roles oral healthcare providers can play in tobacco cessation, more conferences, workshops, and research are needed to motivate and educate oral healthcare providers on tobacco cessation services and develop interventions geared towards dental practices.

## Data Availability

Articles used in this research are available from the authors on reasonable request.
